# Technology-based balance performance assessment can eliminate floor and ceiling effects

**DOI:** 10.1038/s41598-023-41671-8

**Published:** 2023-09-02

**Authors:** Juan Forero, Albert H. Vette, Jacqueline S. Hebert

**Affiliations:** 1 Division of Physical Medicine and Rehabilitation, Department of Medicine, Faculty of Medicine and Dentistry, University of Alberta, Edmonton, AB Canada; 2 Department of Mechanical Engineering, University of Alberta, Edmonton, AB Canada; 3 Glenrose Rehabilitation Hospital, Alberta Health Services, Edmonton, AB Canada

**Keywords:** Outcomes research, Disability

## Abstract

Many clinical measurement tools for balance have ceiling effects. Technology-based assessments using virtual reality systems such as the Computer-Assisted Rehabilitation Environment (CAREN) may provide a way to develop objective, quantitative measures that scale from low to high levels of difficulty. Our objective was to: (1) develop a performance assessment tool (PAT) for the CAREN; (2) quantify the reliability of the tool; (3) validate the scores against clinical balance measures; and (4) compare the scores from a population with balance impairments to those from able-bodied individuals in a cross-sectional validation study. Three games were developed on the CAREN and tested on 49 participants (36 able-bodied and 13 with impaired mobility). For each module, the corresponding measures were transformed into scores using a series of functions such that ceiling and flooring effects would be minimized. The results showed an association between scores and age, an overlap in scores from impaired high-performance individuals and able-bodied low performance individuals, and a correlation of PAT scores with other clinical tests. Several of the limitations of current clinical tools, including floor and ceiling effects, were overcome by the PAT, suggesting that the PAT can be used to monitor the effect of rehabilitation and training.

## Introduction

Balance impairments affect many populations^1–4^. To help affected individuals optimally restore their postural proficiency, rehabilitation professionals can use various clinical assessment tools to obtain information about patients and their rehabilitation progress^5,6^.

Balance performance can be quantified using objective (e.g., time to complete a task) or subjective assessments using an ordinal scoring scale^7^. Functionally relevant clinical tests for assessing balance proficiency fall into one of three categories: timed tests, reaching tests, and stepping tests; these require the patient to perform an activity that involves sitting, standing, or stepping^8^. However, clinical balance assessment tools may show ceiling effects or insensitivity to incremental progress or deterioration in a patient's ability to balance^7,9^ or be unable to differentiate between levels of impairment within a given population^10^. Moreover, these tools focus on measuring a degree of balance proficiency that allows a patient to be discharged from rehabilitation or go back to typical daily life activities. However, this definition of typical activity does not apply to high-performance individuals. For instance, a review of tools available to assess the performance of young, athletic military members with lower-limb amputation showed that affected individuals exceeded the functional expectations set by the outcome measures^11^. In addition, a study reviewing existing test protocols to determine outcomes after a successful rehabilitation showed there is a lack of valid tests to assure a safe reintegration into high performance sports to avoid reinjury^12^. These findings suggest that current tools cannot appropriately assess balance improvements and proficiency in high-performance individuals, such as athletes or military members, which is critical for them to return to high-demand training and duty, respectively.

One attempt to address high-performance needs is through technology-based assessments, training, and interventions. A technology that has recently become popular in rehabilitation paradigms is virtual reality (VR)^13^. VR allows the patient to become immersed in a computer-simulated environment and interact with it naturally and safely^14^. One VR system used for advanced skills training is the Computer-Assisted Rehabilitation Environment (CAREN) developed by Motek Medical (Amsterdam, The Netherlands). The CAREN integrates a movable 6-degree-of-freedom platform, an instrumented treadmill, and a motion capture system with a VR screen^15^. The configurable software allows customization of the virtual environment and its interaction with the user. Research has shown that the CAREN provides a valuable paradigm to expose patients to challenging tasks in a safe environment^16–19^. However, despite this evidence, the unique features of the CAREN have not been exploited in the domain of high-performance balance assessment.

The CAREN system allows the collection of kinematic and kinetic data, making it capable of providing objective and quantitative performance measures. The development of virtual scenarios for postural tasks can accommodate various degrees of difficulty needed for high-performance assessment, potentially overcoming ceiling effects associated with non-adaptable tests. In addition, evidence indicates that VR used during therapy provides a functional, purposeful, and motivating context that promotes patient engagement^20^, which may facilitate the assessment of a patient’s real-world performance. Integrating these features into a high-performance assessment tool would address several limitations that characterize traditional assessment tools.

In the present study, we introduce and evaluate the Performance Assessment Tool (PAT), a novel tool for quantitatively assessing, without ceiling effects, balance performance in high-performing individuals on the CAREN. The objectives of this research were to: (1) develop the PAT as a clinical assessment tool and construct an objective scoring rubric based on the internal consistency of the individual test components; (2) quantify the test–retest reliability of the PAT in an able-bodied population; (3) determine the concurrent, convergent validity of the PAT by comparing its test scores with clinical balance measures in both able-bodied and impaired population samples; and (4) examine the construct validity of the PAT by comparing test scores of a sample population with balance and mobility impairments to able-bodied scores.

## Materials and methods

### Participants

We recruited 49 adult participants from July 2017 to December 2018, with ages ranging between 19 and 66 years (15 female and 34 male). The participants were recruited from a tertiary rehabilitation hospital and through word of mouth. 36 of the participants were able-bodied (AB) (13 females, 23 males, mean age 33 ± 10.1 years) and 13 with impaired mobility (IMP) (2 females, 11 males, mean age 45 ± 15.6 years). Inclusion criteria for those with impaired mobility were: (1) major single lower limb amputation (ankle or more proximal) and wearing a prosthesis for community ambulation; (2) lower limb impairment with permanent musculoskeletal deficiency (i.e., fused joint, peripheral nerve injury) and cleared for full weight bearing with or without bracing support; and (3) diagnosis of traumatic brain injury at least 6 months ago, ambulating in the community. Exclusion criteria for this group were: non-weight bearing on any extremity, unable to walk without walker support, unable to speak English, insufficient capacity for informed consent, or the existence of medical conditions precluding exercise. The resulting IMP cohort was composed of transtibial (n = 7) and transfemoral prosthesis users (n = 4), an individual with a lower limb impairment due to hemophilic arthropathy of the knee (n = 1), and an individual with mild traumatic brain injury (n = 1).

Written informed consent was obtained from all participants, and the experimental procedures were approved by the University of Alberta’s Health Research Ethics Board-Health Panel (Pro00066076) and conducted according to the ethical guidelines. Researchers could identify individual participants during the data collection sessions, but not after the data was collected as each participant was assigned a unique de-identified participant number and only this number was linked to the data.

### Study design

A cross-sectional validation study took place in a tertiary rehabilitation hospital. In addition to one visit for completing the PAT, participants were invited for a second visit to perform standard functional assessment tests in the gymnasium (GYM). Participants from the AB cohort were also invited for a third visit to repeat the PAT on the CAREN. 38 of the 49 total participants completed the GYM testing (29 of the 36 AB and 9 or the 13 IMP), and 13 of the 36 AB participants completed the retest component on the CAREN. The reasons for participants not attending the additional session were related to insufficient time to participate or the inability to contact.

### Experimental procedures

The PAT testing on the CAREN consisted of a series of tasks, repeated for increasing levels of difficulty, grouped within 3 different modules. Completing all the tasks for all levels of difficulty took participants an average of 90 min, ranging between 70 and 120 min. Participants took 3-min breaks in between modules, corresponding to the time it takes the computer to load the application for the next module on the CAREN. Although additional breaks were offered if needed, none of the participants required a longer break. The GYM tests lasted less than 30 min, and participants took 1-min breaks between tasks.

For tests on the CAREN, participants donned a safety harness as per the CAREN protocol and had 3 motion capture marker plates placed: one on each foot, and one on the back at the level of the waist (Supplementary Fig. S4). Before each module started, one of the experimenters explained the process for the module. The participants were informed that a set of instructions would be presented on the screen, describing the goal of the module and the tasks that would follow. Each module consisted of 4 stages: first, the participant was presented with a set of instructions, explaining the task for the module. In some cases, a virtual avatar would demonstrate the task in addition to the visual text instructions. Second, the participant would be asked to assume a comfortable standing position that would be used to calibrate the system. Following calibration, when the participant indicated they were ready, the CAREN operator initialized the first task of the module. At the end of the module, the participant would see a message on the screen to inform them that the module had been completed and the next module will be loaded. At the end of the PAT testing, the participant was guided off the platform, and the motion capture marker plates and safety harness were taken off by an experimenter.

### Task selection

To ensure content validity, we identified a selection of relevant tasks to be implemented on the CAREN for the PAT after examining currently available assessment tools for balance performance in the clinic^7,21^. By deconstructing the tools, we obtained a series of activities that are widely used in balance assessments. Different tasks associated with such activities were then summarized and grouped into 3 separate PAT modules to assess balance performance during: (1) single leg support; (2) stepping in different directions; and (3) standing on a moving platform (perturbations). Each of these modules was presented in the PAT as “games” on the CAREN.

### Task implementation

The 3 different modules developed for the PAT are presented in detail in the Supplementary Information. Module S1 (Supplementary Fig. S1), named “The Blocks”, consists of a game where the participant standing in a virtual field is required to clear the path for an oncoming train of blocks by lifting the foot, thereby challenging single leg stance (*single leg stance*). Module S2 (Supplementary Fig. S2), named “The Targets”, consists of a game where the participant standing in a virtual field is required to use their feet to step on targets appearing in a semicircle in front of them. To succeed, participants had to step with either one or both feet (*challenging stepping*). Module S3 (Supplementary Fig. S3), named “The Bus”, consists of a game where the participant standing inside a virtual bus is required to maintain balance after sudden shifts in the position of the bus, i.e., platform (*perturbation challenge*).

### Outcome measures

Different measures were defined for each module of the PAT according to the task biomechanics within the module:

1. Balance was assessed during standing by measures of the centre of pressure (CoP) displacement^22^. Specifically, the mean velocity of the CoP (*mvCoP*) has been shown to be a reliable measure of balance when the test duration is longer than 20 s^23^. Thus, for “S1: The Blocks”, we computed the outcome measure *mvCoP* from the CoP displacement measured starting the moment the participant’s foot left the ground and ending 30 s later or when the foot was lowered again, whichever occurred first.
2. Foot clearance has been shown to be correlated with stability during walking^24^, and is minimized to save energy at the expense of an increase in the risk of tripping and possibly falling^25^. Conversely, if a risk of falling is perceived, foot clearance usually increases, and a more cautious step will be taken. Under the assumption that a larger foot clearance indicates more cautious stepping, we used the position of the marker clusters on the feet to measure the maximum lift (*peakLift*), defined along the trajectory of the leading foot when stepping onto the targets, as our outcome measure for “S2: The Targets”.
3. Maintaining balance under perturbation conditions relies on different postural strategies depending on the perceived challenge. There are two different classes of postural strategies: (1) “fixed-support” strategies, in which the feet remain in place when responding to the perturbation and balance is maintained using ankle and/or hip motion; and (2) “change-in-support” reactions, where rapid stepping or reaching movements are executed to maintain balance^26^. In general, change-in-support strategies are used when fixed-support responses fail to maintain balance in response to a perturbation. We determined the presence of a stepping response based on the position of the marker clusters on the feet and defined the ratio of the number of trials in which a change-in-support strategy (i.e., stepping response) was necessary over the total number of trials (*ratioStepping*) as our outcome measure for “S3: The Bus”.

### PAT scoring

The scoring for the PAT was defined using data collected on the first visit of participants in the AB cohort. Details on the PAT scoring structure are presented in Supplementary Information.

We defined a series of scoring functions to transform outcome measures into scores. Separate scoring functions were developed for each module based on the module’s corresponding outcome measure. Scoring functions were calculated to fit the data collected from each of the modules. If available, data from both legs were combined for the calculation of the scoring function. Thus, for “S1: The Blocks”, a single scoring function was defined based on values for the measure of *mvCOP* obtained from all participants using the data collected from both legs.

Although, for “S2: The Targets”, measures from both legs were combined, the scoring function was calculated based on additional criteria. First, all but the measures from the diagonal directions (± 45°) were excluded because: (1) for the 0° direction (forward), participants always stepped on the target with the same leg, hence, creating an unbalanced set of data that limits the ability of the measure to characterize the general performance independent of the stepping leg; and (2) the direction of ± 90° (sideways) required a movement that largely limits the movement of the knee; as such, it is not a good measure of general performance. Separate scoring functions were calculated for each combination of level and stepping type (i.e., single foot, double foot). If a target was not reached, then a score of zero was assigned. The scoring functions for “S2: The Targets” were defined based on values for the measure of *peakLift* obtained from all participants for each combination of level and stepping type.

For “S3: The Bus”, separate scoring functions were calculated for each level of difficulty regardless of the direction of the perturbation. The scoring functions for “S3: The Bus” were defined based on values for the measure of *ratioStepping* obtained from each level.

To calculate the combined PAT score, it is necessary to first calculate the scores for each module separately, applying the scoring functions to the measures obtained from the PAT. The scores from S1 and S2 were calculated separately for each leg and the general score was based on the lowest scoring leg. This allowed characterization of the true performance of any given participant. The score for S1 would then be calculated as the lowest score obtained from the individual tests for each leg. For S2, we first calculated the sub-scores for all combinations of level and stepping type separately for each leg, and then calculated, for each leg, the average sub-score. The lowest of these results gives the score for S2. The score for S3 would be calculated as the average of the sub-scores obtained for each level. Finally, the combined PAT score was calculated as the average score across the three modules’ scores.

### Statistical analysis

#### Test–retest reliability

Test–retest reliability was assessed using the subset of participants from the AB cohort that were tested on the CAREN on two separate visits (n = 13). The average time (mean ± SD) between visits was 126 ± 84 days, the minimum being 7 days and the maximum 385 days. Time between sessions was difficult to control due to the challenge of synchronizing the schedule of a given participant with that of CAREN access at the rehabilitation hospital. For the repeat cohort, the Limits of Agreement (LoA) introduced by Bland and Altman^27,28^ were calculated to determine the agreement between the scores (both the individual module scores and the combined PAT scores) from the two different sessions. The LoA also allowed inspecting the results for learning effects between sessions and flagging outlier candidates in the data set^29^. Subsequently, the test–retest reliability of the scores was assessed using both the Intraclass Correlation Coefficient ICC_3,1_^30,31^ and the Concordance Correlation Coefficient CCC, along with its associated bias correction factor C_b_^32,33^.

#### Concurrent convergent validity

Concurrent convergent validity was assessed between the PAT scores from the CAREN and the standard functional assessment tests performed in a gymnasium (GYM). The standard tests used for assessing balance performance in the GYM testing were the Dynamic Gait Index (DGI)^34^, the Berg Balance Scale (BBS)^35,36^, the Amputee Mobility Predictor (AMP)^37^, and the Comprehensive High-Level Activity Mobility Predictor (CHAMP), composed of the Single-Leg Support Test (SLS), the Edgren Side Step Test (ESST), the T-Test (TT), and the Illinois Agility Test (IAT)^38^. For the AMP, only impaired participants were included given the nature of the test (n = 9). For all other tests, able-bodied participants were included in addition to the impaired participants (n = 38). We calculated the Spearman Correlation Coefficient (ρ) to evaluate bivariate correlations between the PAT score and the scores obtained from the DGI, BBS, AMP, SLS, ESST, TT, IAT, and the overall CHAMP.

#### Construct validity

Construct validity of the PAT was examined by inspecting: (1) the ranking of IMP participants compared to AB participants; and (2) the correlation between scores and age. We included data from all cohorts in this study (n = 49). We expected that: (1) participants in the IMP cohort would be ranked at lower levels than participants in the AB cohort; and (2) older participants would be ranked lower than their younger counterparts. We evaluated the Spearman Correlation Coefficient (ρ) between the PAT scores and the participants’ age separately for able-bodied and impaired participants, to account for the inherent difference in performance between both groups.

#### Internal consistency

The internal consistency of the modules of the PAT was inspected to determine the level of correlation across them. Since the three modules evaluate different features of balance performance, we expected them to have some level of agreement, and testing their underlying structure would allow us to determine overlap and redundancy.

We used Cronbach α to determine the internal consistency of the PAT and performed a Principal Component Analysis (PCA) to examine the contribution of each module’s score to the total score.

#### Statistics performed on the PAT scores

The Standard Error of Measurement (SEM) of the combined PAT score was calculated using the intra-class correlation coefficient (ICC_3,1_) and standard deviation (SD) of the PAT score as $SEM=SD\cdot \sqrt{1-{ICC}_{\mathrm{3,1}}}$. The Minimum Detectable Change (MDC) was calculated as $MDC=SEM\cdot z\cdot \sqrt{2}$, with z (z-score) representing the confidence interval for the normal distribution (z = 1.96 for 95% confidence interval)^31^. Differences in the scores for the AB and IMP cohorts were compared by means of a one-way ANOVA. Comparisons were performed on the individual scores for each module as well as on the final combined PAT score.

## Results

**Figure 1:**
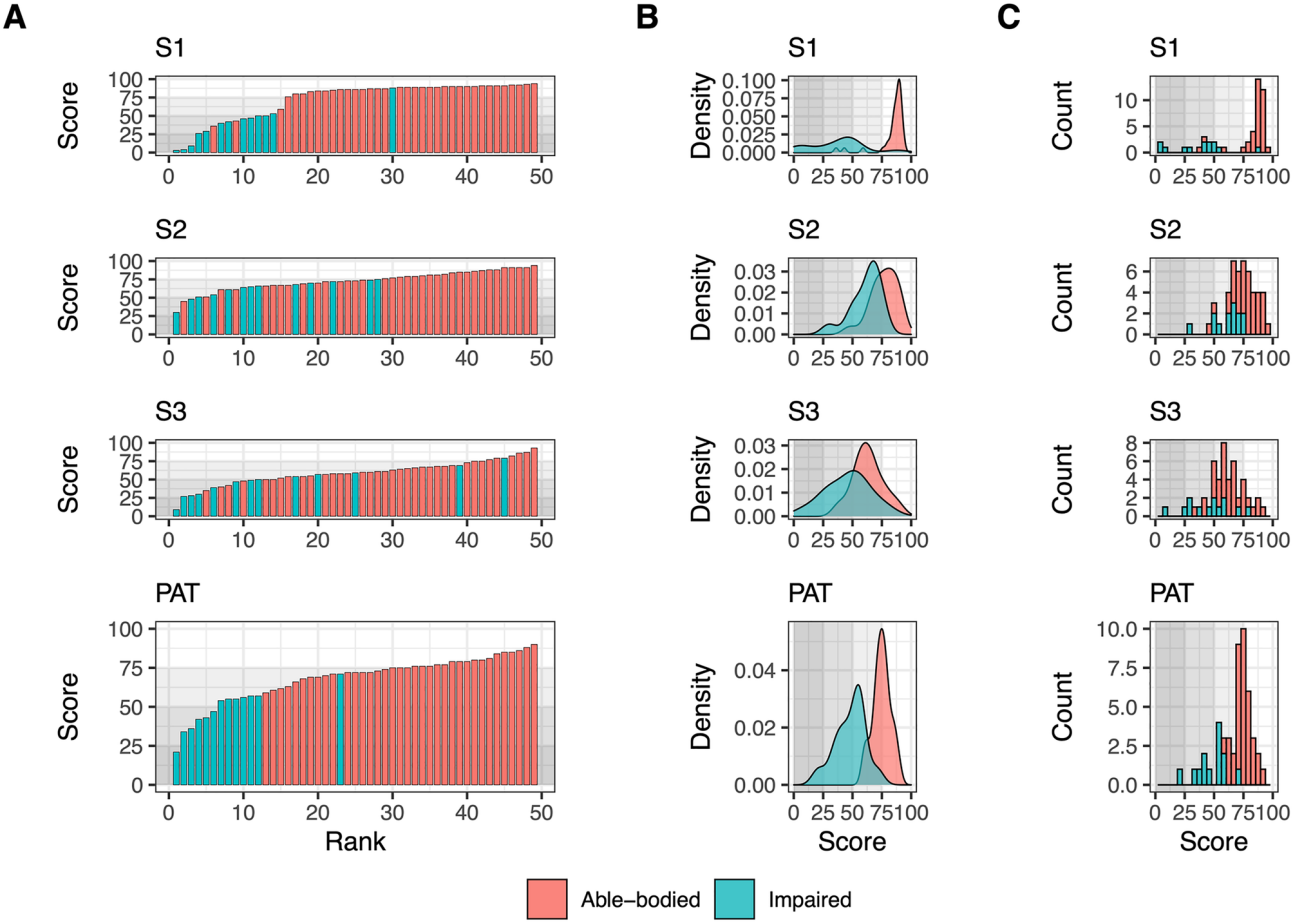
Distribution of the scores obtained by all participants during their first session completing the performance assessment tool (PAT). Scores from the able-bodied cohort are presented in red and scores from the impaired cohort in green. Scores are presented separately for each individual module and for the combined PAT. (**A**) Participant ranking according to their scores. (**B**) Density plot of scores. (**C**) Histogram of individual scores.

Figure 1A presents the scores from the first visit to the CAREN for each participant tested on the PAT, including the separate scores for each module (i.e., S1, S2, S3) and the combined PAT score. The scores are presented sorted by score value to facilitate interpretation. A visual inspection suggests that impaired participants generally scored lower than their able-bodied counterparts, both for the individual modules of the PAT as well as for the entire PAT. This finding is further supported when comparing the distribution of the scores presented in the density plots in Fig. 1B and the histograms in Fig. 1C, where the distribution of scores is discernible between groups. These results are summarized in Table 1. The scores obtained from the able-bodied cohort were found to be significantly higher than those from the impaired cohort as revealed by the one-way ANOVAs calculated for the scores for S1 (F(1,47) = 80.96, p < 0.001), S2 (F(1,47) = 20.30, p < 0.001), S3 (F(1,47) = 12.84, p = 0.001), and the combined PAT score (F(1,47) = 80.65, p < 0.0001). It is important to note that, although some participants scored the minimum score (on S1: lowest score of 0/100) and others close to the maximum score (on S3: highest score of 89/100) on individual modules, none of them scored the minimum or maximum score on the combined score (on PAT: lowest score of 18; highest score of 85). This indicates that the PAT can assess lower as well as higher performers, reducing the possibility of flooring and ceiling effects on the combined score.

**Table 1:** Descriptive statistics for the tests on the PAT.

	S1 “The Blocks”	S2 “The Targets”	S3 “The Bus”	PAT
Able-bodied				
Mean ± SD	72.0 ± 17.5	64.8 ± 11.2	57.4 ± 11.6	64.8 ± 7.8
Minimum	29	43	35	54
25th percentile	69	58	50	59
50th percentile	79	63	56	66
75th percentile	83	70	63	68
Maximum	89	84	86	85
Impaired				
Mean ± SD	29.1 ± 23.1	50.5 ± 11.0	45.9 ± 19.0	42.2 ± 12.4
Minimum	0	23	9	18
25th percentile	9	44	30	36
50th percentile	37	54	49	46
75th percentile	39	57	57	50
Maximum	81	63	79	65

**Figure 2:**
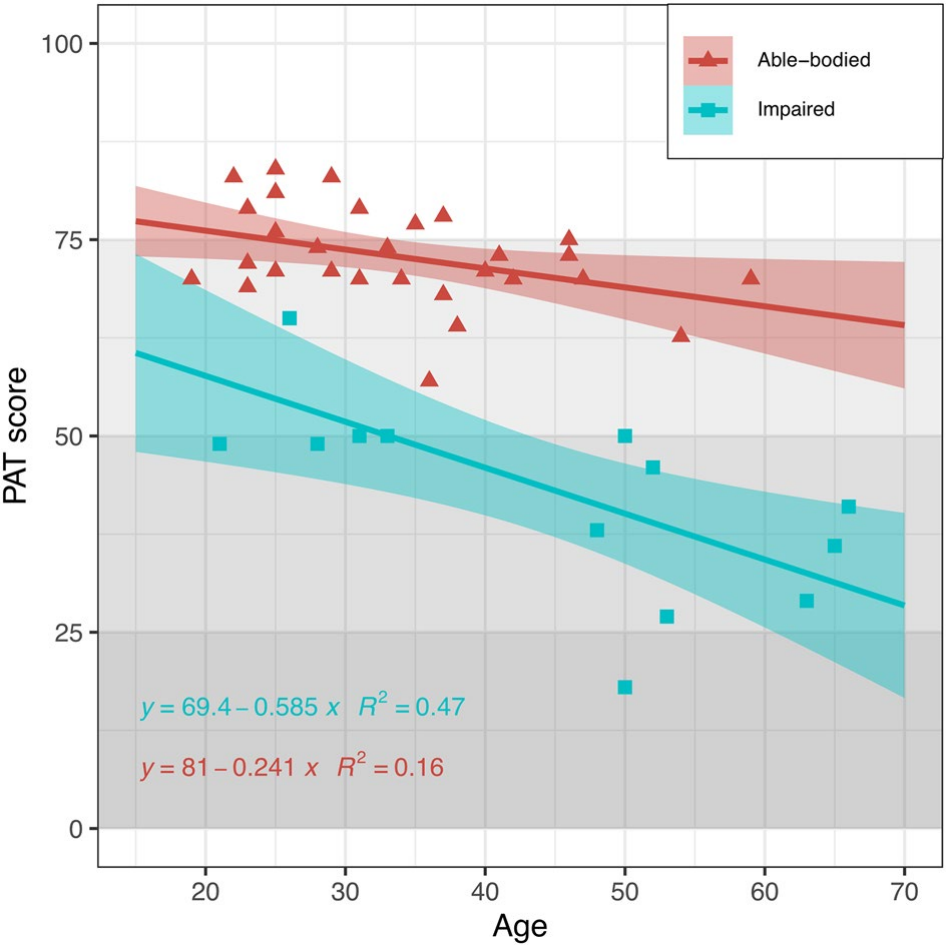
Scatter plot showing the correlation between age (in years) and the performance assessment tool (PAT) score. Data are presented separately for able-bodied and impaired participants. A linear trend is presented for each set, together with a 95% confidence band.

In addition to comparing the ranking based on the PAT scores between able-bodied and impaired participants, we also related the PAT scores to the age of the participants. Figure 2 shows the age correlation with the scores obtained. There was a general trend for the PAT score to decrease with age. Overall, scores from impaired participants are lower than those from able-bodied participants across all ages, and scores from younger participants are higher than those from older ones.

To evaluate the test–retest reliability of the PAT, we estimated the LoA to verify the agreement of the repeat testing results and to inspect for outliers. The Bland–Altman plot (Supplementary Fig. S5) suggested that testing scores between sessions were in high agreement as most of the points were tightly packed within the 95% confidence band around the mean. Only data from one participant showed low agreement between visits and was flagged as an outlier. Data from that participant (marked with an X in Supplementary Fig. S5) were excluded from the calculations of the test–retest reliability. The average of the mean differences between scores from the first and second visit was negative across all modules (average of mean differences: S1: − 3.79; S2: − 3.50; S3: − 8.29; PAT: − 5.36), suggesting the presence of a learning effect between sessions. The results for ICC_3,1_, CCC, C_b_ and α are reported in Table 2. The test–retest reliability for S1 (ICC_3,1_ = 0.956) was high, and although that of S2 (ICC_3,1_ = 0.569) and S3 (ICC_3,1_ = 0.694) was moderate, the one resulting from combining the three scores into the PAT was high (ICC_3,1_ = 0.826). These results were further confirmed by the results for CCC (S1: 0.953; S2: 0.759; S3: 0.545; PAT: 0.707) and C_b_ (S1: 0.996; S2: 0.951; S3: 0.784; PAT: 0.855). The internal correlation measured by α showed a high correlation for all scores (S1: 0.977; S2: 0.865; S3: 0.829; PAT: 0.969).

**Table 2:** ICC, CCC and Cronbach correlation coefficients.

	S1 “The Blocks”	S2 “The Targets”	S3 “The Bus”	PAT
Intraclass correlation				
ICC_3,1_	0.956	0.569	0.694	0.826
95% CI	0.864–0.987	0.054–0.846	0.256–0.895	0.524–0.943
Concordance correlation				
CCC	0.953	0.759	0.545	0.707
95% CI	0.854–0.985	0.053–0.827	0.142–0.793	0.365–0.882
C_b_	0.996	0.951	0.784	0.855
Cronbach correlation				
α	0.977	0.865	0.829	0.969
95% CI	0.948–0.990	0.689–0.942	0.604–0.926	0.929–0.987

**Figure 3:**
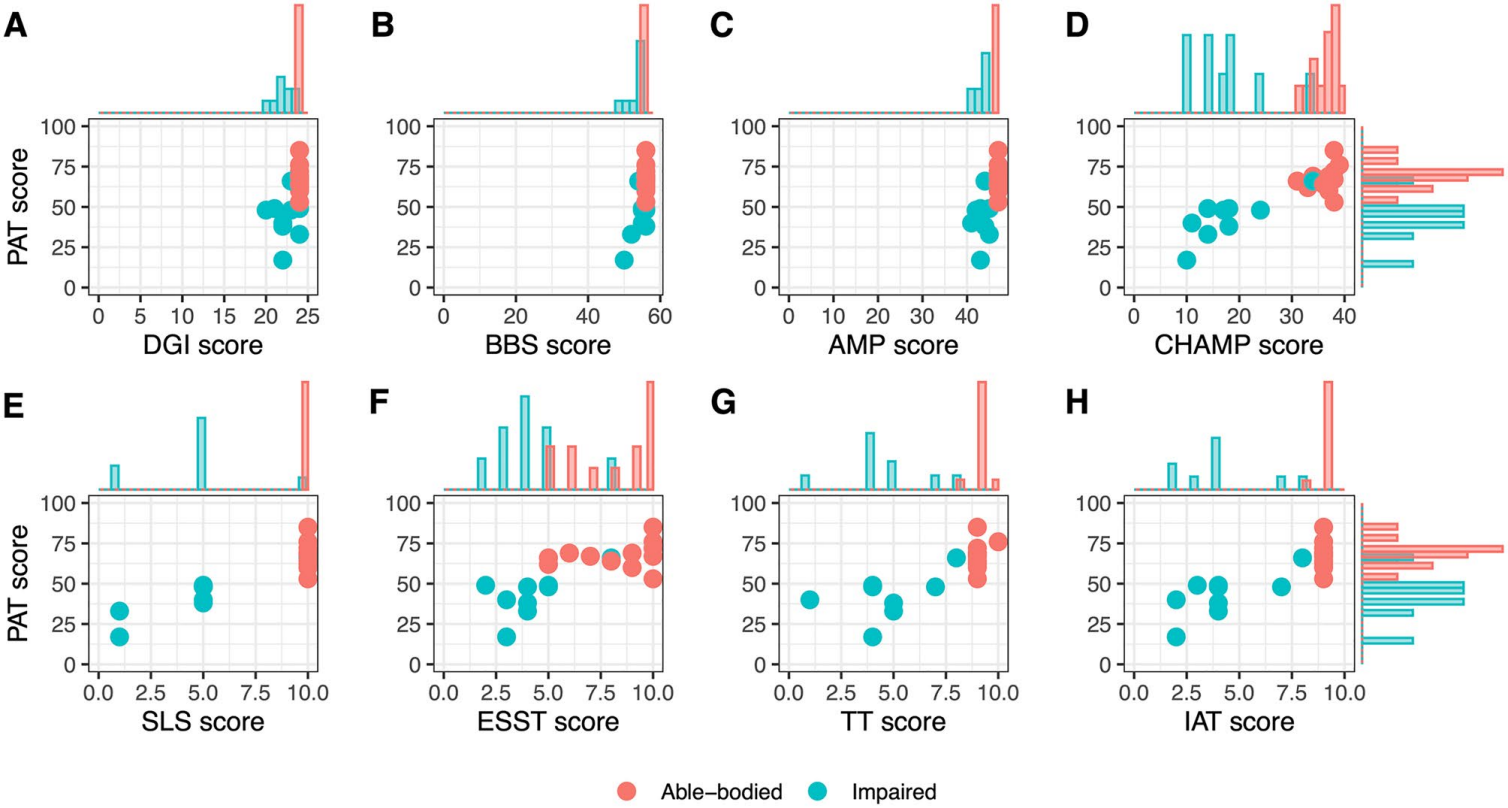
Marginal plots with histograms comparing the distribution of the scores from all tests. Comparisons are presented for the performance assessment tool (PAT) scores for the able-bodied and impaired individuals against (**A**) the Dynamic Gait Index (DGI), (**B**) the Berg Balance Scale (BBS), (**C**) the Amputee Mobility Predictor (AMP), and (**D**) the combined score for the Comprehensive High-Level Activity Mobility Predictor (CHAMP) as well as (**E**) the Single-Leg Support Test (SLS), (**F**) the Edgren Side Step Test (ESST), (**G**) the T-Test (TT), and (**H**) the Illinois Agility Test (IAT), i.e., the individual scores for the CHAMP. Histograms are presented for the scores on each of the standard tests above the scatter plots and on the right side of the right-most plots (**D**,**H**) for the PAT test.

The results from the standard functional tests included in this study are summarized in Table 3, and the comparison of these scores to those obtained with the PAT is presented in Fig. 3. The marginal plots presented for the scores DGI, BBS, and AMP show the limitation of these tests to differentiate participants according to their performance. For these tests, scores from both the AB and IMP cohorts are tightly clustered towards the highest values, revealing a clear ceiling effect across participants. In comparison, there is a greater spread of results of the PAT for both AB and IMP cohorts. For the AB cohort, a similar ceiling effect can also be seen for SLS, TT, and IAT. Although a different behaviour is seen for the IMP cohort in these tests, i.e., showing a better spread of the scores, the scores seem to cluster into groups. A different result is observed for the scores from ESST and CHAMP. The scores from these tests show a better spread across participants for both the AB and IMP cohorts. While the scores from the ESST and CHAMP are well spread across a wide range for both cohorts, they still present a ceiling effect with many participants from the AB cohort scoring the maximum. Conversely, the scores from the PAT present a well-spread range of scores for both cohorts, without reaching either the maximum or minimum values, hence leaving still room for higher as well as lower performers to be characterized.

**Table 3:** Scores across all GYM^a^ outcome measures for IMP and AB participants.

	DGI	BBS	AMP	SLS	ESST	TT	IAT	CHAMP
Able-bodied								
Mean ± SD	24 ± 0.0	56 ± 0.0	47 ± 0.0	10 ± 0.0	8.1 ± 2.0	9.0 ± 0.4	8.9 ± 0.3	36.0 ± 2.4
Minimum	24	56	47	10	5	8	8	31
25th percentile	24	56	47	10	6	9	9	34
50th percentile	24	56	47	10	9	9	9	37
75th percentile	24	56	47	10	10	9	9	38
Maximum	24	56	47	10	10	10	9	39
Impaired								
Mean ± SD	22 ± 1.3	54 ± 2.1	43 ± 1.3	4.7 ± 2.6	4.2 ± 1.7	4.7 ± 2.0	4.2 ± 2.1	17.8 ± 7.4
Minimum	20	50	41	1	2	1	2	10
25th percentile	22	54	43	5	3	4	3	14
50th percentile	22	55	44	5	4	4	4	17
75th percentile	23	56	44	5	5	5	4	18
Maximum	24	56	45	10	8	8	8	34

^a^Tests performed on the gymnasium (GYM) visit: Dynamic Gait Index (DGI), Berg Balance Scale (BBS), Amputee Mobility Predictor (AMP), Single-Leg Support Test (SLS), Edgren Side Step Test (ESST), T-Test (TT), Illinois Agility Test (IAT), and the combined score for the Comprehensive High-Level Activity Mobility Predictor (CHAMP).

**Figure 4:**
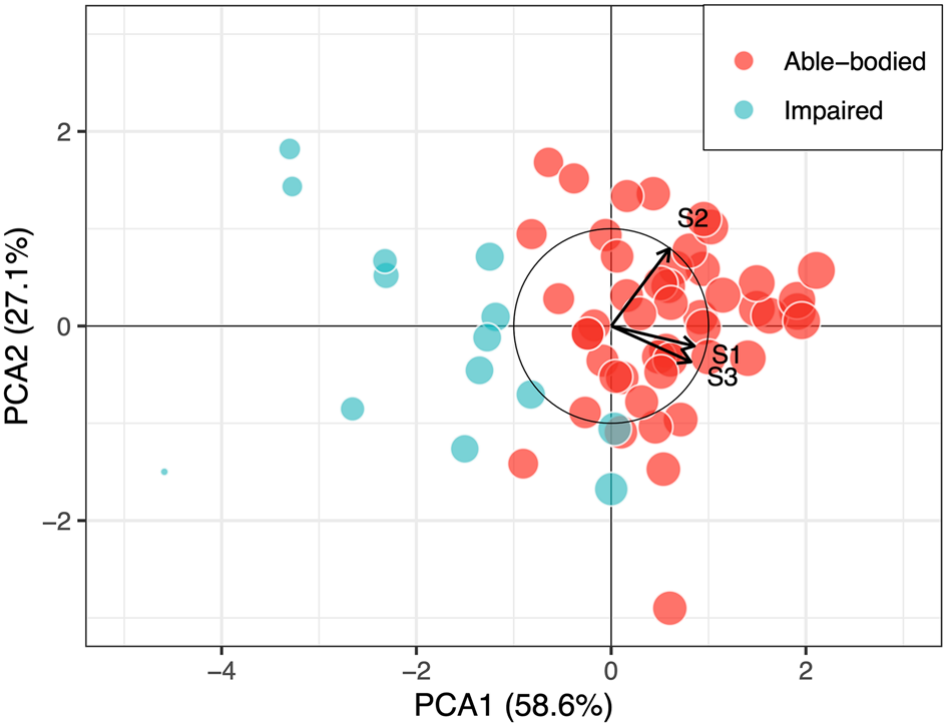
PCA biplot for the PAT score. The biplot shows the principal component analysis (PCA) scores of the participants’ PAT scores (green circles for impaired participants and red circles for able-bodied participants) as well as the loading of the module scores (black vectors, one for each of the module scores). PC scores are presented for the two main components, which together accounted for 85.7% of the variability (PCA1 accounted for 58.6% and PCA2 for 27.1% of the total variability). The size of the circles is scaled to the magnitude of the PAT score for comparison.

The results obtained from the PCA on the PAT data from both AB and IMP cohorts are summarized in Table 4. The first two principal components account for 86.1% (PC1: 59.8%; PC2: 26.3%) of the variability of the data. The biplot in Fig. 4 presents the PAT scores from the first visit, with the horizontal axis representing the first principal component (PC1) and the vertical axis the second principal component (PC2). As depicted by the principal component loadings presented in Table 4 and denoted in Fig. 4 by the S1, S2, and S3 arrows pointing in the positive direction of PC1, the scores from all three modules of the PAT are positively correlated with the dominant component (i.e., PC1) of the PAT score. The scores from S1 and S3 are highly correlated with each other but have a low correlation with the score from S2. In particular, S1 and S3 contribute strongly to PC1 and PC3 while S2 substantially contributes to PC2. From the component loadings one can see that the contribution of S1 and S3 to PC1 (0.853 and 0.818, respectively) and PC2 (− 0.206 and − 0.369, respectively) are similar; however, their contribution to PC3 is opposite (− 0.479 and 0.441, respectively). In addition, the features measured by S1 and S3, pointing in the negative direction of PC2, differ from those measured by S2, pointing in the opposite direction along PC2.

**Table 4:** PCA results summary.

	PC1	PC2	PC3
Component loadings			
S1	0.853	− 0.206	− 0.479
S2	0.600	0.796	0.080
S3	0.818	− 0.369	0.441
Component explained variation			
PAT	59.8%	26.3%	13.9%

Another important observation from the PCA is the ability for the PAT score to discriminate scores from able-bodied participants from those from impaired participants: the scores for able-bodied participants are clustered toward the right (positive PC1), and the ones for impaired participants are clustered toward the left (negative PC1). Both sets of data share a small common area of overlap around the midline (PC1 = 0), reflecting the scores from low-performance able-bodied participants and high-performance impaired participants.

The SEM for the PAT was calculated using the combined PAT score test–retest reliability coefficient (ICC_3,1_: r = 0.826) and SD (7.8 points). The identified SEM for the PAT was 3.3 points. The MDC for the PAT was calculated using the z-score for 95%CI (z_95_ = 1.96), the combined PAT score test–retest reliability coefficient (ICC_3,1_: r = 0.826), and SD (7.8 points). The MDC for the PAT was 9.0 points.

## Discussion

We developed a clinical assessment tool capable of quantifying balance performance changes without the limitations associated with ceiling or flooring effects. The PAT was developed by creating a series of activities inspired by current knowledge on balance performance assessment and combining them into separate modules presented as virtual games to the users. By presenting the tasks as games, users were encouraged to perform them naturally so that their quantified performance would represent their typical movements. Balance and performance scores on the PAT were built on objective measurements based on the biomechanics of bipedal balance^6–8^. The conversion functions from measures into scores were developed so that the maximum score is only reached with a perfect performance, and the lowest score is never reached and only approached asymptotically, thus eliminating ceiling and flooring effects.

The overall scores show an overlap between the scores from impaired participants and those from able-bodied participants (Fig. 1A). The overlap is linked to the presence of high-performance impaired participants as well as low-performance able-bodied participants. Although the individual module scores present an overlap across cohorts, which hinders a clear separation between the groups, the combined PAT score produces a much better differentiation (Fig. 1B). The score distribution from able-bodied participants is shifted to the right compared to impaired participants, showing a trend for able-bodied participants to score higher than impaired participants. Although both density plots expose a symmetrical distribution, the scores from impaired participants have a larger spread than that from able-bodied participants, reflecting greater variability. However, even with both groups showing large spreads in the scores, there is enough room in both directions to address higher or lower scores, reflecting performance improvement or worsening without foreseeing a ceiling or flooring effect: the highest scores obtained by the tested cohorts do not reach the maximum value, nor do the lowest scores reach the minimum value.

The most visible improvement from the PAT over the comparison tests is overcoming the ceiling effects where high performers will saturate the scale^11^. Ceiling effects present in the scores from the DGI, BBS and AMP (tests designed for impaired participants) were not seen with the PAT. The PAT was also compared to the CHAMP, a measure designed to assess high-performance individuals within the military^38^. The CHAMP presented a clear cut-off between cohorts, contrary to the PAT, which presented a less robust differentiation of the groups with more overlap between cohorts. However, the PAT presented a wide spread in the scores obtained by impaired participants, reflecting the ample range of performance, where high-performing impaired participants could be distinguished from other impaired participants. The ability to distinguish levels of impairment within participants has been pointed out as a limitation of standard clinical measures^10^. Our approach with the PAT was to deliver a tool that would allow following the progress of users of any level of performance (from very low to high). The approach with the PAT allows its use not only for assessing progress during rehabilitation in impaired participants but also assessing progress towards higher level fitness or performance goals due to the lack of flooring or ceiling effects.

The PAT scores showed a trend of a decreased score with age (Fig. 2) in both cohorts. Moreover, the decrease in performance seemed steeper for the impaired compared to the able-bodied cohort, as reflected by the slope of the linear fit. It is known that the mechanisms involved in postural control become less effective with advancing age^39^. Although the neural components associated with the reduction of balance performance are various^40^, the result is a decrement in balance performance with age. Interestingly, our results suggest that the PAT captures not only the effect of aging on changes in performance in the able-bodied population but also in the presence of impairment.

The PAT was tested for repeatability by testing participants on two different days. However, due to the difficulties in synchronizing the participants’ schedule with CAREN availability, the time between visits was inconsistent across participants and, in some cases, too long (up to a year between visits). The effect of this issue was not observed in the results from the correlation test on the PAT, showing it to be a reliable test (Table 2). Although the high agreement in the Bland-Altmann plots suggests the PAT tool to be reliable, a second observation must be made (Supplementary Fig. S5): the negative value for the mean of the difference indicates that the scores from the second visit were consistently higher than those of the first visit. The able-bodied cohort had an estimated difference of about 10 points between visits (Fig. 2). Such difference could be attributed to a learning effect related to participants getting familiarized with the testing process^41^. Also, because participants can be overwhelmed by the CAREN system the first time they see it, the differences in performance observed between visits could be partly attributed to familiarity with the CAREN system. It is important to note that, even in the presence of such differences, the reliability of the PAT was still high and that the average observed difference in scores between visits was very close to the calculated MDC of 9.0 points for the PAT.

An important feature of combining scores from different tests is ensuring the measurements do not overlap. Due to the nature of the balance activities, one score could overlap with another, making it unnecessary to have multiple tests and instead reduce the number of tests without losing the strength of the final measure. From the ranking plot for all three sub-scores, the score from S1 dominated the clustering between cohorts, as the scores from S2 and S3 indicate an overlap between groups (Fig. 1A). However, the results from the PCA show the contribution of S2 to the final score to be independent of the one of S1 and S3. Moreover, neither of the sub-scores correlates directly with the main component (Table 4, coefficients for PC1), but rather the final score is a combination of all the scores.

It could be argued that, although S1 and S3 use different tasks, they measure similar features of balance that could overlap. Indeed, both the task in S1 and S3 evaluate balance performance due to perturbations during standing: S1 measures balance in response to internal perturbations (i.e., how balance is maintained while standing on one leg), and S3 measures balance in response to external perturbations. The component loadings for S1 and S3 show that their contribution to PC1 and PC2 are comparable, but their contribution to PC3 is opposite (Table 4). Although this could be assumed to show the importance of including both tests, the difference in their contribution to the final score is rather small as PC3 only accounts for 13.9% of the total score. Thus, one could suggest that an appropriate assessment could be made by having S2 combined with either S1 or S3.

Moreover, the reliability of the score from S3 when calculated separately was lower than that of S1, S2, and the combined PAT score (Table 2). The reason for including S3 within the PAT was that it encompasses the reactive component of balance, which seemed highly relevant but might not play a strong role when assessing balance performance. It would then be encouraged to eliminate S3 from the battery and, with it, eliminate the need for the CAREN. Consequently, a “reduced PAT” could be implemented outside of the CAREN, facilitating the tool's implementation.

### Limitations

The current study had three main limitations. First, the testing protocol requires time to instrument participants by donning the markers and setting them up on the CAREN system for a total testing time that might be considered long for a clinical assessment. The added time might also result in fatigue of the participants, affecting their performance. As mentioned above, a shortened PAT could be designed to reduce the time burden. Another limitation results from access to the technology, as the CAREN system is only available in a select number of rehabilitation centres. However, using this assessment to study impaired participants could be efficiently done in larger rehabilitation centres with access to a CAREN to provide valuable information on the efficacy of balance interventions. A third limitation can be pointed out with respect to the number of injured participants included in the study. To better evaluate the performance of the tool, larger groups with similar diagnosis would better illuminate differences in abilities specific to each group.

## Conclusion

The performance assessment tool (PAT) presented and evaluated in this study addressed several limitations that characterize current assessment tools. The PAT provides a gaming environment where users will be more engaged during the tasks; hence, measurements may better correlate with optimal capabilities. Moreover, the instructions required for the user to perform the tasks are easier to understand and follow due to the nature of the games. Finally, since the scoring is adjusted to eliminate flooring and ceiling effects, the PAT can be used to monitor not only low-balance performers during their rehabilitation, but also high-performance users and their improvement associated with training. Although the CAREN is not accessible to everyone, the games developed for the PAT can be translated to other setups that are easier to accommodate in the clinic. Future research could use the PAT to examine the efficacy of balance rehabilitation interventions, expand the capabilities of the PAT, and assess balance during more challenging tasks such as walking.

### Supplementary Information

Supplementary Information.

## Data Availability

The datasets generated during and/or analysed during the current study are available from the corresponding author on reasonable request.
