# Supplementary material for: Technology-based balance performance assessment can eliminate floor and ceiling effects
Source: Sci Rep. 2023 Sep 2;13:14488. doi: 10.1038/s41598-023-41671-8 (PMC10475013; doi:10.1038/s41598-023-41671-8)
Supplement: Supplementary file 1 — Supplementary Information. [file 41598_2023_41671_MOESM1_ESM.pdf]

Technology-based balance performance assessment can eliminate floor and ceiling effects
# - Supplementary Information -

Modules/Games development

Three modules were created in the form of games for the PAT to assess balance performance during 1) single leg support, 2) stepping in different directions, and 3) standing on a moving platform (perturbations). Details on the development of such modules are presented below.

1) The Module S1, named "The Blocks" (S1 Fig), consists of a game where participants standing in a virtual field are required to clear the path for an oncoming train of blocks by lifting one foot. Each train of blocks consists of one-meter-long blocks. Blocks are released on the ground from a distance of 5 m and approach the participant's feet at a constant speed of 1 m/s. The blocks can approach from either the right or left sides, requiring the participant to lift the corresponding leg. Trains of different lengths (1, 5, 15 and 30 m) were used in this task, requiring different lifting times from the participant. The time required for the blocks to pass under either foot, from the moment the blocks reached the foot until the moment they had all passed, was 1, 5, 15, and 30 seconds for each of the train lengths. If the foot is not lifted high enough to clear the blocks, these would be destroyed, and a *FAILURE* auditory sound would be played. Conversely, if the blocks are safely cleared then a *SUCCESS* sound is played. Blocks were first presented on the right side and then on the left.

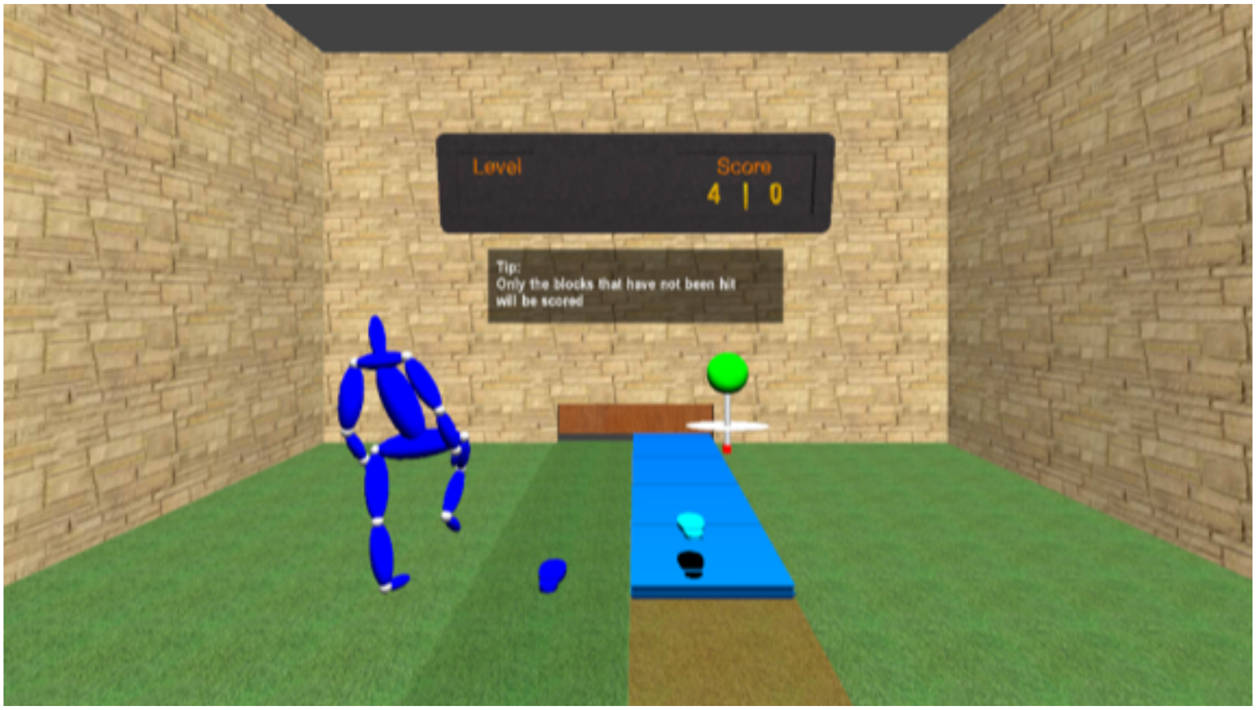

Supplementary Figure S1. Screenshot from the interface for the Module S1.

2) The Module S2, named "The Targets" (S2 Fig), consists of a game where participants standing in a virtual field are required to use their feet to pop targets appearing in front of them. Targets would appear in one of 5 directions (N, NW, NE, W, E) and require the participant to reach targets with one foot or step on them with both feet. Participants are not required to use a specific foot to contact the targets but need to maintain contact for at least 2 seconds for the target to pop and a message asking them to go back to the initial standing posture to appear. Targets will appear at different distances measured from the participant's feet. Four different levels of challenge were defined by the distance at which targets appear, these being 30%, 50%, 70% and 90% of the participant's leg length for level 1, level 2, level 3 and level 4 respectively. Due to the size restriction on the CAREN platform, targets appeared in all 5 directions for levels 1 through 3, but only in 3 directions (N, NW and NE) for level 4.

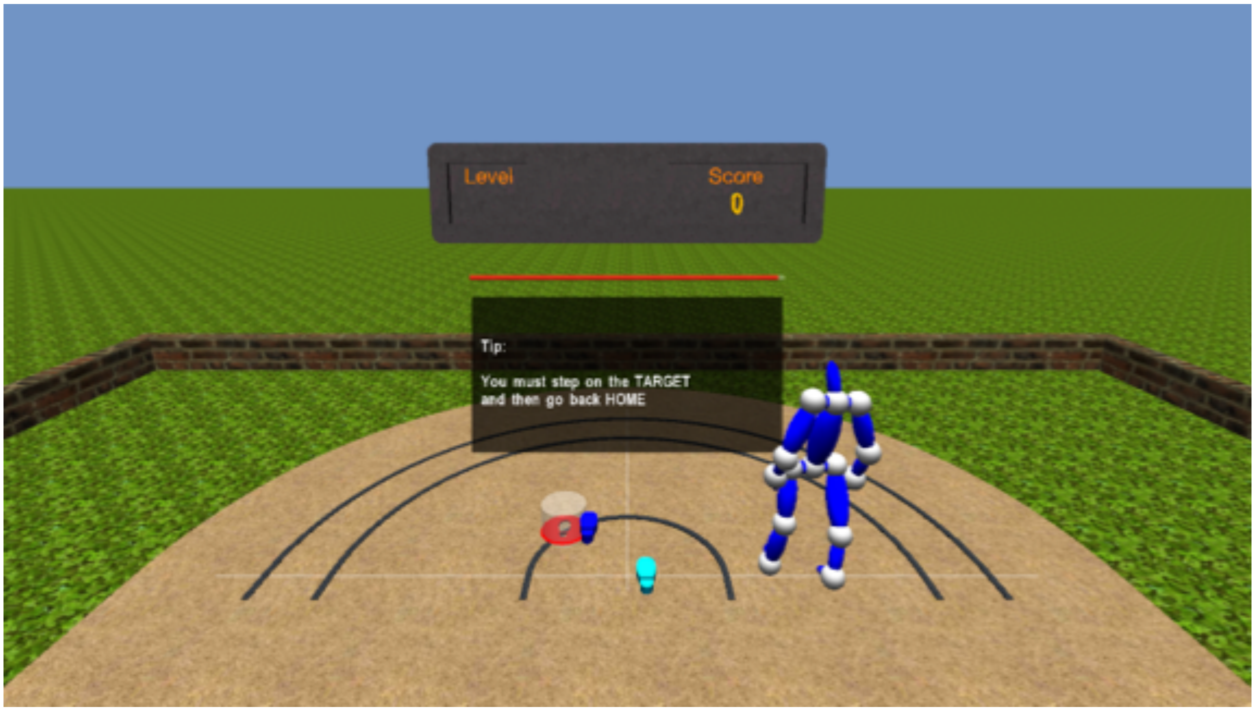

Supplementary Figure S2. Screenshot from the interface for the Module S2.

3) The Module S3, named "The Bus" (S3 Fig), consists of a game where participants standing inside a virtual bus are required to maintain balance after sudden shifts in the position of the platform. The platform will rapidly shift in one of 8 different directions (N, S, W, E, NW, NE, SW, SE) with different accelerations. Four different levels of challenge were defined by the acceleration of the platform movement, quantified by the time required for the platform to reach a displacement of 20 cm upon delivery of the perturbation. The times associated with each level were of 2400 ms, 1600 ms, 800 ms, and 400 ms. Participants are required to maintain balance during the movement of the bus without using any additional support. The task is repeated for all directions, moving away from the center and then back, for 4 levels of difficulty, each based on the acceleration of the platform described above.

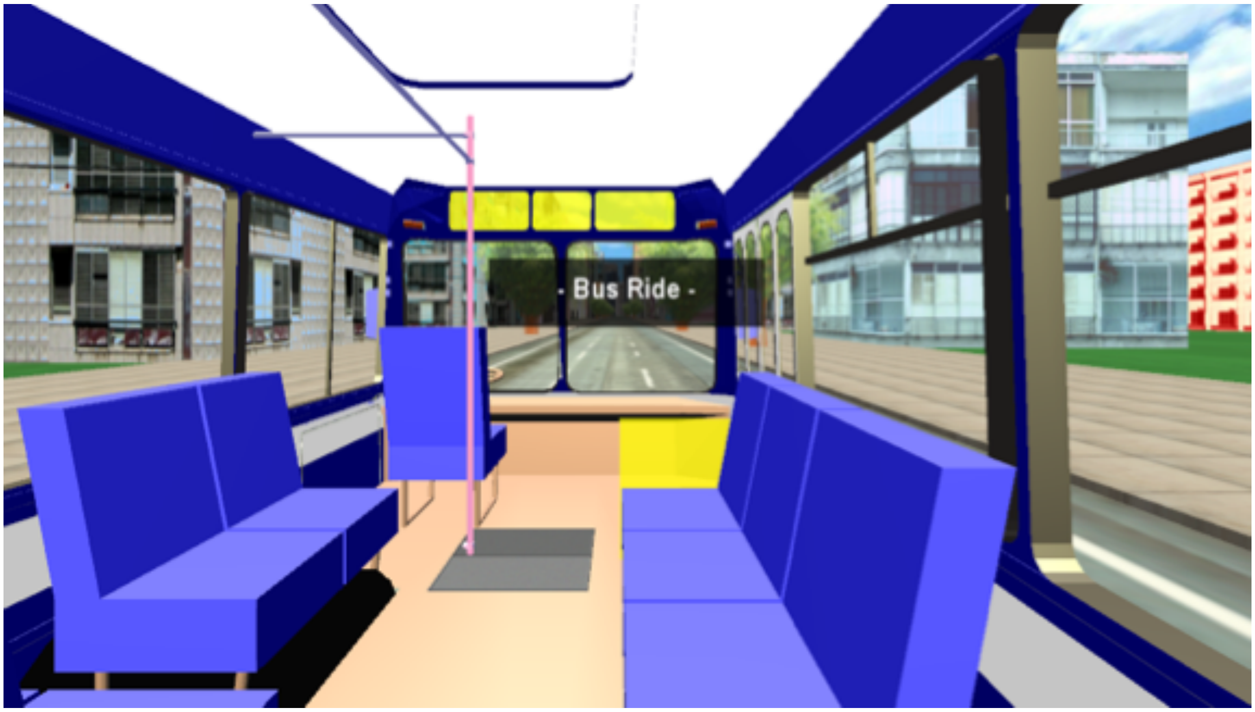

Supplementary Figure S3. Screenshot from the interface for the Module S3.

The interaction between the participants and the games was measured by tracking markers placed on the feet and the back at the level of the waist (S4 Fig). Markers in the feet were used to track the movement of the feet in each of the aforementioned games. The markers in the back were used to estimate the length of the leg for each participant and scale the visual feedback from the games accordingly. Markers were tracked using the 12- camera motion capture system (Vicon Inc; Oxford, United Kingdom) integrated into the CAREN.

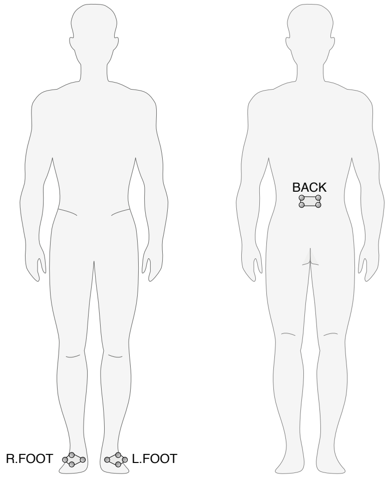

Supplementary Figure S4. Marker placement to track participants' interaction with the games.

PAT scoring structure

Separate scores were calculated for each leg (when available) within each module based on the module's corresponding outcome measures. For "S1: The Blocks", separate scores were calculated for each foot depending on which foot was required to be lifted during each task. For "S2: The Targets", separate scores were also calculated for each foot, depending on the stepping foot. In addition, separate scores were also calculated for each level of difficulty. For "S3: The Bus", separate scores were calculated for each level. The structure for the definition of all sub scores and their relation to each module as well as to the final score is presented in **Supplementary Table S1**.

Supplementary Table S1. Games' structure depicting the leg being assessed, levels and scores used to generate game scores and the final score.

Module	Level	Leg	Leg Score	Level Score	Module Score	Final Score
S1: The Blocks	Level 1	Left	S1_L	S1_0 = min(S1_L, S1_R)	S1_score = S1_0	PAT_score = mean(S1_score, S2_score, S3_score, S4_score)
		Right	S1_R			
S2: The Targets	Level 1	Left	S2_1_L	S2_1 = min(S2_1_L, S2_1_R)	S2_score = mean(S2_1, S2_2, S2_3, S2_4)	
		Right	S2_1_R			
	Level 2	Left	S2_2_L	S2_2 = min(S2_2_L, S2_2_R)		
		Right	S2_2_R			
	Level 3	Left	S2_3_L	S2_3 = min(S2_3_L, S2_3_R)		
		Right	S2_3_R			
	Level 4	Left	S2_4_L	S2_4 = min(S2_4_L, S2_4_R)		
		Right	S2_4_R			
S3: The Bus	Level 1	-		S3_1	S3_score = mean(S3_1, S3_2, S3_3, S3_4)	
	Level 2	-		S3_2		
	Level 3	-		S3_3		
	Level 4	-		S3_4		

Conversion functions between outcome measures and task scores were defined separately for each combination of task and level within each game. For these functions, the following rules were set:

a) all score values range between 0 and 100,
b) a score value of 0 is associated with the lowest possible performance for a task,
c) a score value of 100 is associated with the highest possible performance for a task,
d) a score value of 70 is associated with the 95th percentile for the corresponding measure,
e) the conversion function is defined only for positive values of the measures,
f) the function for converting measures into scores is defined by a polynomial,
g) the conversion function approaches the score of zero asymptotically.

Given the choice of measures, for all task measures in all three modules a flawless performance is associated with the measure value of zero and a score of 100. Conversely, for the lowest performance measure values, the following definitions were used:

a) a score of zero was defined for any value of *mvCOP* higher than 0.5 m/s,
b) a score of zero was defined for any value of *peakLift* higher than 20 cm,
c) a score of zero was defined for a value of *ratioStepping* of 100%.

The resulting transformation functions were of the form:

$$Score = 100 - a \cdot Measure + b \cdot Measure^2 - c \cdot Measure^3$$

For “S1: The Blocks”, separate measures were obtained for each leg. A single scoring function (Eq. 1) was defined using the best-performing leg, i.e., using the lower of the two values for the measures of *mvCOP* obtained from each participant. If the obstacles are not cleared then a score of zero will be assigned.

For “S2: The Targets”, separate scoring functions were defined for each combination of direction and stepping type (i.e., single foot, double foot), but only the data collected from the lowest level (i.e., level 1) was used in the definition of the scoring function. All but the measures from the diagonal directions (±45˚) were excluded because 1) the direction for 0˚ allowed participants to use either foot, limiting the ability of the measure to characterize the performance for each foot; and 2) the direction of ±90˚ requires a movement that largely limits the movement of the knee; as such, it is not a good measure of general performance. Similar to “S1: The Blocks”, separate measures were obtained for each leg, and the final score was calculated using the measure from the best-performing leg, i.e., the lowest of the two values for the measure of *peakLift*. The final score for “S2: The Targets” was calculated as the average of the scores across levels and stepping types. If a target is not reached, then a score of zero will be assigned.

For “S3: The Bus”, a single scoring function was defined for the measures obtained from the lowest level (i.e., level 1). The final score for “S3: The Bus” was calculated as the average of the scores across levels.

The resulting coefficients for each of the scoring function are presented in Table S2.

Finally, to calculate the combined PAT score, we averaged the individual scores from each module.

Supplementary Table S2. Scoring function coefficients.

Module	Measure	Type	Scoring function parameters	Function
S1: The Blocks	mvCOP (in m/s)	-	a = -528.0474<br>b = 912.1895<br>c = -512.1895	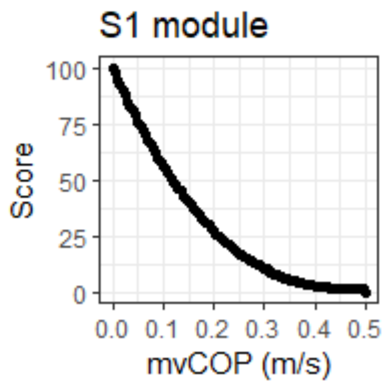
S2: The Targets	peakLift (in m)	1 Foot	a = 208.1829<br>b = -9581.8291<br>c = 30204.5726	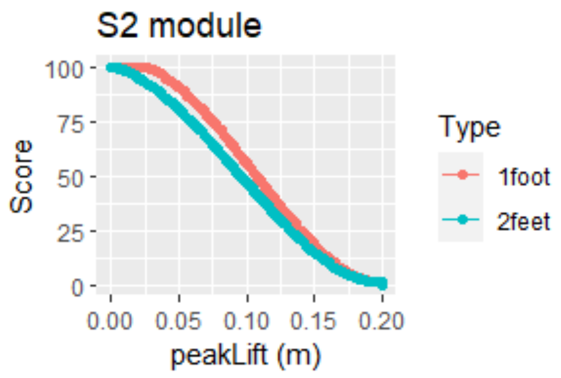
		2 Feet	a = -150.3489<br>b = -5996.5107<br>c = 21241.2767	
S3: The Bus	#stepping responses	Level 1	a = -7.1825<br>b = -0.1278<br>c = 0.0121	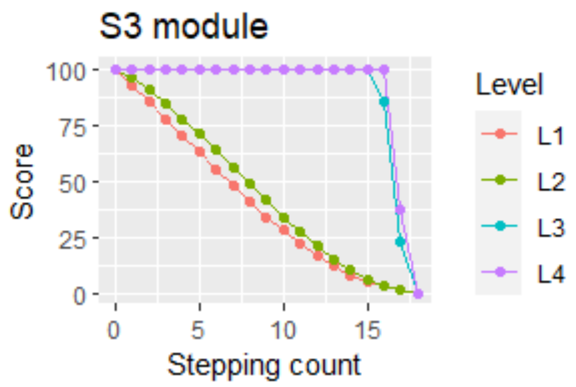
		Level 2	a = -3.9954<br>b = -0.4819<br>c = 0.0219	
		Level 3	a = 414.8148<br>b = -47.0164<br>c = 1.3145	
		Level 4	a = 679.9388<br>b = -76.4746<br>c = 2.1328	

Additional figures

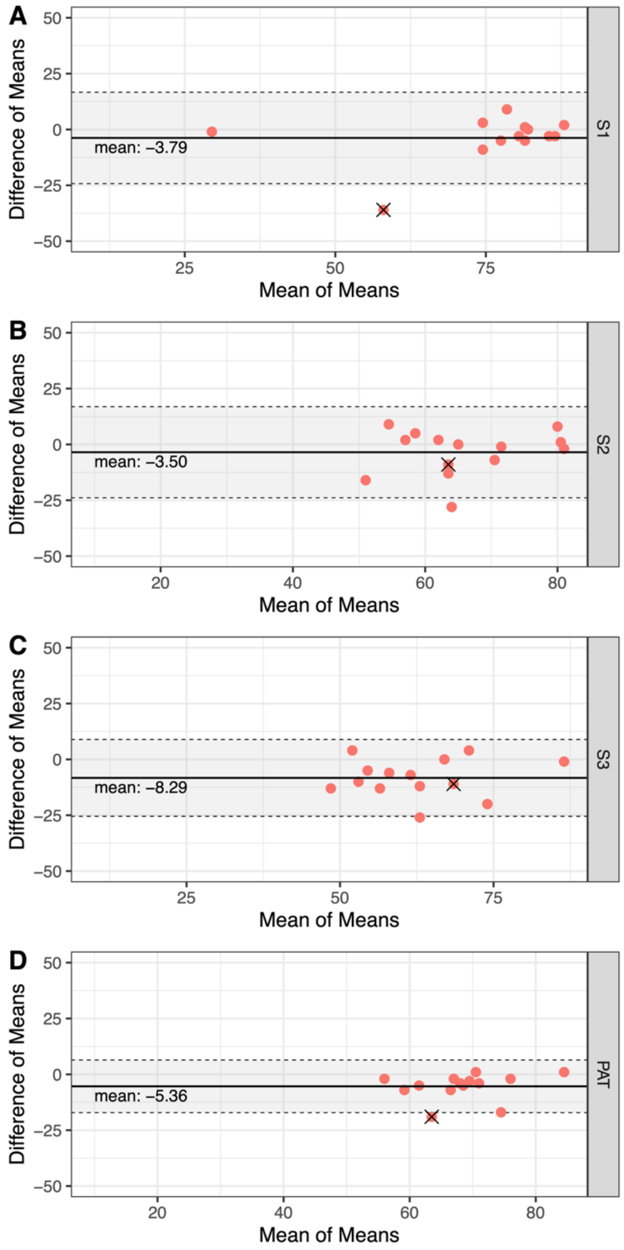

Supplementary Figure S5. Bland-Altman plot comparing testing scores between sessions.
